# Supplementary material for: Immunological Effects of an Add-On Physical Exercise Therapy in Depressed Adolescents and Its Interplay with Depression Severity
Source: Int J Environ Res Public Health. 2021 Jun 17;18(12):6527. doi: 10.3390/ijerph18126527 (PMC8296386; doi:10.3390/ijerph18126527)
Supplement: Supplementary file 1 [file ijerph-18-06527-s001.zip › ijerph-1233785-supplementary.pdf]

International Journal of
Environmental Research and Public Health

MDPI

E-Supplements:

Table S1. Study Design

**Screening**		
Inpatient treatment<br>Major Depression (SKID I)<br>DIKJ> 18 raw points<br>No exclusion criteria<br>Meeting Inclusion criteria		
**3 Measure Points**	**Physical Measures**:	**Psychological Parameters:**
t0=Inclusion<br><br>t1= after 6 weeks intervention<br><br>t2= 8 weeks after t1 (no further intervention)	Spiroergometry including lactate blood levels<br><br>Jump mechanography<br><br>Calipermetry, BMI	Clinical interview (SKID I)<br><br>Depression questionnaires (DIKJ, BDI II)<br><br>Sport questionnaires (Motivation and Barriers to Sports, MSES)<br><br>Feed-Back questionnaire
**1 Post Measurement**<br><br>Post t2 =<br>3 months after t2 (no further intervention)		Depression questionnaires (DIKJ, BDI II)

See: Wunram et al., Eur Child Adolesc Psychiatry. 2018 May;27(5):645-662.

Table S2. Exercises of WBV-Training and Ergometer Training

**WBV: Exercise description**	frequency/ amplitude	Sessions 1-12	Sessions 13 onward	**Ergometer training**	
				Intervals in minutes	% of maximal wattage in spiroergometry
"See-Saw": Rocking the feet between the ball and the heel	20 Hz/ 2	2 min./2 min. pause	3 min./ 3 min. pause	3 min.	40%
Squats: approximately 20 per minute	20 Hz/ 2	2 min. ./2 min. pause	3 min. ./ 3 min. pause	6 min.	50 %
„Tree in the wind": Lateral side-movement with outstretched arms above the head	20 Hz/ 2	2 min. ./2 min. pause	3 min. ./ 3 min. pause	3 min.	70/80 %
„Rotation": Lateral trunk-rotation of horizontally extended arms from left to right	20 Hz/ 2	2 min. ./2 min. pause	3 min. ./ 3 min. pause	6 min.	50 %
„Holding arms": Arms 90 degree extended in front of the body and pulling a theraband	20 Hz/ 2	2 min. ./2 min. pause	3 min. ./ 3 min. pause	3 min.	70/80 %
„The Chair": holding posture with 90 degrees bended knees like if sitting on a virtual chair	20 Hz/ 2	2 min. ./2 min. pause	3 min. ./ 3 min. pause	6 min.	50 %
				3 min.	40 %

See also: Wunram et al., Eur Child Adolesc Psychiatry. 2018 May;27(5):645-662.

Table S3. Therapies (in Minutes)

Length of stay in days therapy time in minutes	Total [51]	Ergometer [16]	WBV [18]	Control [17]	p
Total therapy week 1-6	1077 (419)	1149 (419)	979 (381)	1113 (463)	0.691
Total therapy week 7-14	410 (583)	606 (784)	268 (314)	375 (566)	0.147
Psychotherapy week 1-6	311 (173)	311 (157)	309 (178)	314 (193)	0.954
Psychotherapy week 7-14	108 (167)	190 (243)	71 (105)	70 (104)	0.026
Art therapy week 1-6	304 (191)	294 (161)	314 (255)	304 (143)	0.598
Art therapy week 7-14	122 (203)	173 (230)	89 (153)	109 (224)	0.359
Sports therapy week 1-6	240 (266)	256 (258)	161 (171)	308 (339)	0.474
Sports therapy week 7-14	97 (182)	117 (227)	42 (66)	134 (212)	0.324
Group therapy week 1-6	32 (51)	35 (41)	26 (40)	36 (71)	0.958
Group therapy week 7-14	16 (51)	17 (49)	5 (17)	26 (73)	0.568
Music therapy week 1-6	36 (78)	39 (113)	26 (55)	43 (58)	0.522
Music therapy week 7-14	10 (46)	32 (127)	9 (40)	1 (4)	0.457
Ergo therapy week 1-6	11 (72)	32 (127)	0	4 (18)	0.391
Ergo therapy week 7-14	0	0	0	0	-
Social Service week 1-6	55 (73)	62 (78)	54 (60)	12 (23)	0.796
Social Service week 7-14	29 (69)	41 (102)	26 (55)	43 (58)	0.364
Parents meetings week 1-6	86 (84)	120 (83)	89 (94)	52 (61)	0.024
Parents meetings week 7-14	28 (54)	48 (75)	16 (42)	22 (34)	0.061

Mean (SD); p from one-way ANOVA; [N].

Table S4. Pairwise Comparisons of Estimated Marginal Means and Standard Errors (SE) for IL-6 and TNF-α Measures for all Treatment Groups Over Time

Treatment group	Time (I)	Time (J)	IL-6 med			IL-6 max			IL-6 min			TNF-α		
			Mean Diff. (I-J)	SE	p-value	Mean Diff. (I-J)	SE	p-value	Mean Diff. (I-J)	SE	p-value	Mean Diff. (I-J)	SE	p-value
Ergometer	baseline	6 weeks	0.570	0.328	0.086	0.353	0.281	0.213	0.784	0.397	0.052	0.124	0.179	0.490
		14 weeks	0.916	0.423	0.033	0.600	0.338	0.079	1.237*	0.525	0.020	-0.042	0.228	0.853
WBV	baseline	6 weeks	0.177	0.325	0.588	0.131	0.279	0.640	0.226	0.394	0.568	-0.142	0.182	0.439
		14 weeks	-0.115	0.410	0.779	-0.303	0.328	0.358	0.075	0.507	0.883	-0.070	0.227	0.758
Control	baseline	6 weeks	0.594	0.350	0.094	0.462	0.297	0.124	0.711	0.427	0.100	0.198	0.191	0.302
		14 weeks	0.193	0.456	0.673	0.057	0.361	0.876	0.319	0.567	0.576	0.133	0.271	0.625

IL-6 and TNF-α values reported in pg/ml.

Table S5. TNF-α Over Time for Gender

**Descriptive Statistics TNF-α means**				
	Gender	Mean	SD	N
TNF-α t0	male	2.0164	0.64647	13
	female	1.4332	0.64274	29
	total	1.6138	0.69203	42
TNF-α t1	male	2.0644	1.07757	13
	female	1.3374	0.57602	29
	total	1.5625	0.82592	42
TNF-α t2	male	2.2742	1.19535	13
	female	1.3491	0.52151	29
	total	1.6354	0.88957	42

Table S6. Descriptives Spiroergometry and Leonardo Mechanography

Group		T0 maxWatt/KG	T1 maxWatt/KG	T2 maxWatt/KG	T0 RERpeak	T1 RERpeak	T2 RERpeak	T0 Jump peak Watt/KG	T1 Jump peak Watt/KG	T2Jump peak/Watt/KG
Ergometer	Mean	1.81	2.17	2.13	24.59	25.78	29.95	38.73	38.39	39.80
	SD	0.58	.61	0.65	7.96	6.59	6.55	9.25	7.87	7.14
	Minimum	0.70	1.08	1.26	12.55	14.66	21.18	18.10	22.43	30.98
	Maximum	3.08	3.49	3.52	38.77	42.31	41.62	56.37	56.50	56.59
WBV	Mean	2.01	1.97	1.89	26.78	26.23	26.25	37.63	38.12	37.49
	SD	0.56	.63	0.55	7.76	6.92	8.69	6.87	6.59	6.68
	Minimum	1.01	0.99	1.07	13.03	16.01	14.92	27.38	28.19	29.34
	Maximum	3.08	3.44	3.36	39.76	43.03	44.20	52.69	48.36	49.46
Control	Mean	1.89	1.90	1.90	24.64	25.43	25.93	36.91	37.99	38.03
	SD	0.52	0.53	0.51	8.27	7.15	5.93	8.17	9.55	10.36
	Minimum	0.84	1.21	0.89	5.53	13.88	14.52	25.17	24.26	24.37
	Maximum	3.09	3.25	3.18	42.03	42.13	41.15	58.87	60.85	59.73
Total	Mean	1.90	2.02	1.97	25.35	25.84	27.41	37.71	38.17	38.42
	SD	0.55	0.60	0.58	7.95	6.75	7.32	8.04	7.87	7.88
	Minimum	0.70	0.99	0.89	5.53	13.88	14.52	18.10	22.43	24.37
	Maximum	3.09	3.49	3.52	42.03	43.03	44.20	58.87	60.85	59.73

See also: Wunram et al., Eur Child Adolesc Psychiatry. 2018 May;27(5):645-662.

Table S7. Influences of Covariates on IL-6 med, Estimates of Fixed Effects

Parameter	Estimate	SE	*p*-value	95% Confidence Interval	
				Lower Bound	Upper Bound
Intercept	0.70	1.62	0.667	−2.55	3.95
[Group=0]	−0.92	0.77	0.235	−2.45	0.61
[Group=1]	−0.58	0.73	0.426	−2.03	0.86
[Group=2]	0[b]	0	.	.	.
[Time=1]	0.09	0.46	0.845	−0.82	1.00
[Time=2]	−0.45	0.39	0.257	−1.24	0.34
[Time=3]	0[b]	0	.	.	.
[Time=1] * [Group=0]	0.91	0.64	0.157	−0.36	2.18
[Time=2] * [Group=0]	0.84	0.54	0.122	−0.23	1.91
[Time=3] * [Group=0]	0[b]	0	.	.	.
[Time=1] * [Group=1]	−0.16	0.62	0.802	−1.39	1.08
[Time=2] * [Group=1]	0.17	0.52	0.738	−0.86	1.21
[Time=3] * [Group=1]	0[b]	0	.	.	.
[Time=1] * [Group=2]	0[b]	0	.	.	.
[Time=2] * [Group=2]	0[b]	0	.	.	.
[Time=3] * [Group=2]	0[b]	0	.	.	.
[Sex=0]	−0.09	0.21	0.674	−0.51	0.33
[Sex=1]	0[b]	0	.	.	.
[Medication=0]	0.84	0.27	0.003	0.31	1.38
[Medication=1]	0.11	0.36	0.754	−0.61	0.84
[Medication=2]	0.57	0.65	0.384	−0.74	1.89
[Medication=3]	0[b]	0	.	.	.
Age	−0.03	0.09	0.782	−0.22	0.167
BMI	0.09	0.01	0.000	0.06	0.12
Number of trainings	0.02	0.03	0.418	−0.03	0.07
Total therapy time 6Weeks	−0.00	0.00	0.082	−0.00	5.32
Total therapy time 8Weeks	−0.00	0.00	0.449	−0.00	0.00

a. Dependent Variable: IL6 med; b. This parameter is set to zero because it is redundant. SE= standard error.

Table S8. Influences of Covariates on IL-6 max, Estimates of Fixed Effects

Parameter	Estimate	SE	*p*-value	95% Confidence Interval	
				Lower Bound	Upper Bound
Intercept	2.40	1.14	0.040	0.12	4.67
[Group=0]	−0.52	0.57	0.362	−1.64	0.61
[Group=1]	−0.16	0.54	0.768	−1.22	0.91
[Group=2]	0b	0	.	.	.
[Time=1]	−0.03	0.35	0.934	−0.72	0.67
[Time=2]	−0.40	0.31	0.202	−1.01	0.22
[Time=3]	0b	0	.	.	.
[Time=1] * [Group=0]	0.66	0.49	0.177	−0.30	1.63
[Time=2] * [Group=0]	0.69	0.42	0.108	−0.155	1.5
[Time=3] * [Group=0]	0b	0	.	.	.
[Time=1] * [Group=1]	−0.26	0.47	0.588	−1.20	0.68
[Time=2] * [Group=1]	−0.03	0.40	0.950	−0.84	0.78
[Time=3] * [Group=1]	0b	0	.	.	.
[Time=1] * [Group=2]	0b	0	.	.	.
[Time=2] * [Group=2]	0b	0	.	.	.
[Time=3] * [Group=2]	0b	0	.	.	.
[Sex=0]	0.03	0.14	0.835	−0.26	0.32
[Sex=1]	0b	0	.	.	.
[Medication=0]	0.58	0.18	0.003	0.21	0.95
[Medication=1]	0.08	0.26	0.739	−0.43	0.60
[Medication=2]	−0.05	0.45	0.919	−0.95	0.86
[Medication=3]	0b	0	.	.	.
Age	−0.07	0.07	0.294	−0.21	0.06
BMI	0.06	0.01	0.000	0.04	0.08
Total number of trainings	0.00	0.02	0.847	−0.03	0.04
Total therapy time 6 weeks	−0.00	0.00	0.108	−0.00	5.94
Total therapy time 8 weeks	−9.69	0.00	0.453	−0.00	0.00

a. Dependent Variable: IL-6 max. b. This parameter is set to zero because it is redundant. SE=standard error.

Table S9. Influences of Covariates on IL-6 min, Estimates of Fixed Effects

Parameter	Estimate	SE	*p*-value	95% Confidence Interval	
				Lower Bound	Upper Bound
Intercept	−0.97	2.27	0.673	−5.53	3.60
[Group=0]	−1.21	1.02	0.237	−3.24	0.81
[Group=1]	−0.91	0.96	0.349	−2.82	1.00
[Group=2]	0b	0	.	.	.
[Time=1]	0.21	0.59	0.723	−0.96	1.38
[Time=2]	−0.48	0.50	0.333	−1.48	0.51
[Time=3]	0b	0	.	.	.
[Time=1] * [Group=0]	1.16	0.81	0.158	−0.46	2.78
[Time=2] * [Group=0]	0.99	0.67	0.149	−0.36	2.34
[Time=3] * [Group=0]	0b	0	.	.	.
[Time=1] * [Group=1]	−0.07	0.79	0.934	−1.63	1.50
[Time=2] * [Group=1]	0.36	0.65	0.587	−0.95	1.66
[Time=3] * [Group=1]	0b	0	.	.	.
[Time=1] * [Group=2]	0b	0	.	.	.
[Time=2] * [Group=2]	0b	0	.	.	.
[Time=3] * [Group=2]	0b	0	.	.	.
[Sex=0]	−0.22	0.29	0.462	−0.81	0.38
[Sex=1]	0b	0	.	.	.
[Medication=0]	1.09	0.38	0.007	0.32	1.86
[Medication=1]	0.143	0.51	0.780	−0.88	1.16
[Medication=2]	1.16	0.94	0.223	−0.74	3.07
[Medication=3]	0b	0	.	.	.
Age	0.02	0.13	0.877	−0.25	0.29
BMI	0.11	0.02	0.000	0.071	0.16
Total number of trainings	0.03	0.03	0.322	−0.03	0.10

Total therapy time 6 weeks	−0.00	0.00	0.099	−0.00	0.00
Total therapy time 8 weeks	−0.00	0.00	0.489	−0.00	0.00

a. Dependent Variable: IL-6 min. b. This parameter is set to zero because it is redundant. SE= standard error.

Table S10. Influences of Covariates on TNF-α, Estimates of Fixed Effects

Parameter	Estimate	SE	*p*-value	95% Confidence Interval	
				Lower Bound	Upper Bound
Intercept	2.56	1.57	0.111	−0.62	5.73
[Group=0]	−0.46	1.22	0.709	−2.92	2.00
[Group=1]	−0.43	1.17	0.716	−2.79	1.93
[Group=2]	0[b]	0	.	.	.
[Time=1]	0.18	0.27	0.500	−0.35	0.71
[Time=2]	−0.12	0.24	0.597	−0.60	0.35
[Time=3]	0[b]	0	.	.	.
[Time=1] * [Group=0]	−0.15	0.35	0.670	−0.85	0.55
[Time=2] * [Group=0]	0.04	0.31	0.898	−0.58	0.66
[Time=3] * [Group=0]	0[b]	0	.	.	.
[Time=1] * [Group=1]	−0.25	0.35	0.468	−0.94	0.44
[Time=2] * [Group=1]	0.20	0.30	0.515	−0.41	0.81
[Time=3] * [Group=1]	0[b]	0	.	.	.
[Time=1] * [Group=2]	0[b]	0	.	.	.
[Time=2] * [Group=2]	0[b]	0	.	.	.
[Time=3] * [Group=2]	0[b]	0	.	.	.
[Sex=0]	0.82	0.20	0.000	0.41	1.24
[Sex=1]	0[b]	0	.	.	.
[Medication=0]	0.29	0.25	0.258	0.080	0.80
[Medication=1]	0.35	0.3439	0.312	−0.34	1.03
[Medication=2]	−0.59	0.61	0.342	−1.82	0.65
[Medication=3]	0[b]	0	.	.	.
Age	−0.12	0.10	0.236	−0.31	0.08
BMI	0.02	0.01	0.125	−0.01	0.05
Total number of trainings	0.01	0.05	0.772	−0.08	0.11
Total therapy time 6 weeks	0.00	0.00	0.625	−0.00	0.00
Total therapy time 8 weeks	−8.10	0.00	0.653	−0.00	0.00

a. Dependent Variable: TNF-α. b. This parameter is set to zero because it is redundant. SE= standard error.

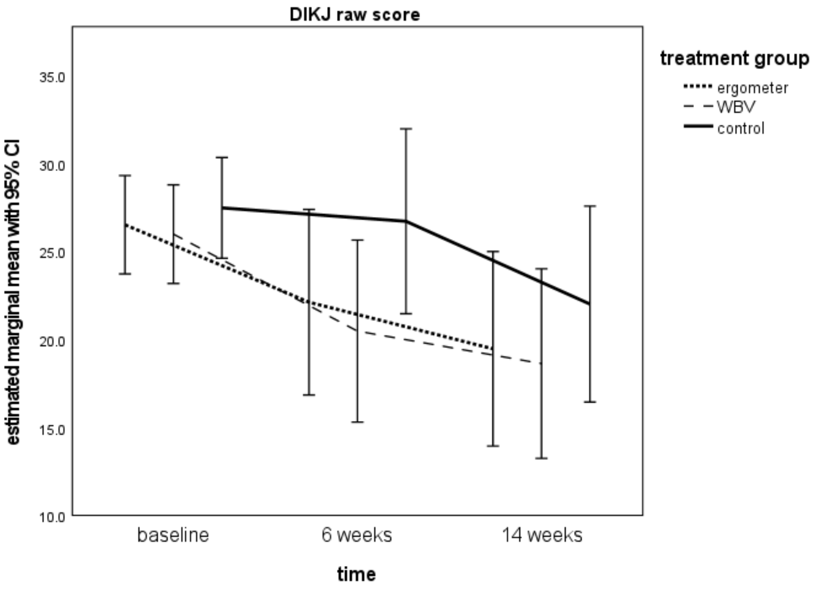

Figure S1. Mean-DIKJ raw score mixed model analysis. See: Wunram et al., Eur Child Adolesc Psychiatry. 2018 May;27(5):645-662.

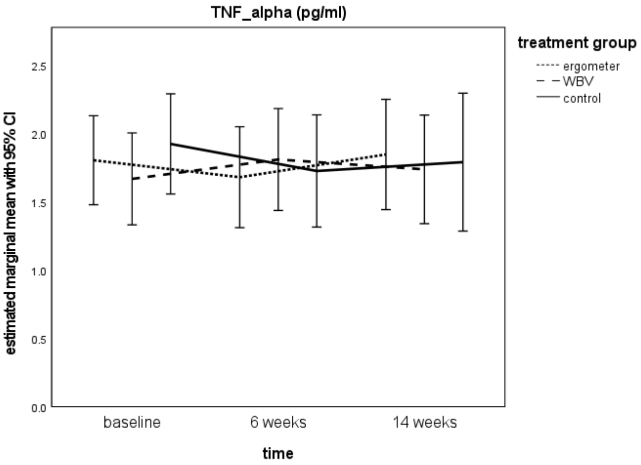

S2

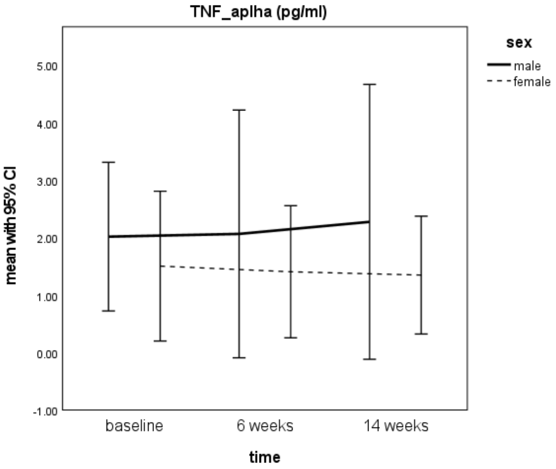

S3

Figure S2. and S3: TNF-α means and gender differences between groups over time (TNF-α values reported in pg/ml).
